# Alkaloid Quantities in Endophyte-Infected Tall Fescue are Affected by the Plant-Fungus Combination and Environment

**DOI:** 10.1007/s10886-016-0667-1

**Published:** 2016-01-27

**Authors:** M. Helander, T. Phillips, S. H. Faeth, L. P. Bush, R. McCulley, I. Saloniemi, K. Saikkonen

**Affiliations:** Department of Biology, University of Turku, 20014 Turku, Finland; Department of Plant and Soil Sciences, University of Kentucky, Lexington, KY 40546-0312 USA; Department of Biology, University of North Carolina, Greensboro, NC 27402-6170 USA; Natural Resources and Biomass Production Research, Natural Resources Institute Finland (Luke), 20520 Turku, Finland

**Keywords:** Ergot alkaloids, Fungal endophytes, Grasses, Lolines, Lysergic acid, Nutrients, Water

## Abstract

Many grass species are symbiotic with systemic, vertically-transmitted, asymptomatic *Epichloë* endophytic fungi. These fungi often produce alkaloids that defend the host against herbivores. We studied how environmental variables affect alkaloids in endophyte-infected tall fescue (*Schedonorus phoenix*) from three Northern European wild origins and the widely planted US cultivar ‘Kentucky-31’ (KY31). The plants were grown in identical common garden experiments in Finland and Kentucky for two growing seasons. Plants were left as controls (C) or given water (W), nutrient (N) or water and nutrient (WN) treatments. For 8–10 replications of each plant origin and treatment combination in both experiments, we analyzed ergot alkaloids, lysergic acid, and lolines. In Finland, tall fescue plants produced 50 % more ergot alkaloids compared to plants of the same origin and treatments in Kentucky. Origin of the plants affected the ergot alkaloid concentration at both study sites: the wild origin plants produced 2–4 times more ergot alkaloids than KY31, but the ergot alkaloid concentration of KY31 plants was the same at both locations. Overall lysergic acid content was 60 % higher in plants grown in Kentucky than in those grown in Finland. Nutrient treatments (N, WN) significantly increased ergot alkaloid concentrations in plants from Finland but not in plants from Kentucky. These results suggest that the success of KY31 in US is not due to selection for high ergot alkaloid production but rather other traits associated with the endophyte. In addition, the environmental effects causing variation in alkaloid production of grass-endophyte combinations should be taken into account when using endophyte-infected grasses agriculturally.

## Introduction

Grasses (Poaceae) have invaded every continent of the globe, and cover more terrestrial area than any other group of plants. Temperate grasses of the subfamily Pooideae are integral components of natural grassland ecosystems. These grasses also are economically important crop plants that are cultivated in monocultures and polycultures, mainly as animal forage to help feed the globe’s growing population.

The majority of grass species in pastures and natural grasslands are perennials, capable of tolerating high grazing pressure from vertebrate herbivores. This tolerance is based mainly on their rapid regrowth capacity, their underground storage organs, and their ability to grow new tillers following defoliation (Dyer et al. 1991; Karban and Baldwin 2007). The dominance of grassland ecosystems across all the continents except Antarctica indicates that grasses are competitive across a range of abiotic conditions. Yet grasses, despite high levels of herbivory, appear to invest little in chemical defense compared to other plants. Grasses typically have high levels of silicon (Si) phytoliths in leaf and other above ground parts, and these are thought to provide physical defense against herbivory (Huitu et al. 2014; Rudall et al. 2014; Vicari and Bazely 1993). The ability of grasses to produce secondary chemical defense compounds such as alkaloids is limited (Huitu et al. 2014; Vicari and Bazely 1993).

Instead of producing chemical compounds themselves, many pooid grasses have coevolved with vertically-transmitted, symbiotic *Epichloë* fungi (Saikkonen et al. 2013; Schardl et al. 2013) whose asexual forms formerly were referred to as *Neotyphodium* (Leuchtmann et al. 2014). These hereditary symbioses are typically host specific. The fungi grow in intercellular spaces in the above-ground parts of grasses. *Epichloë* endophytes in their host grasses are capable of producing four classes of alkaloids, which may provde the endophyte-grass symbiota with defenses against herbivores. The ergot alkaloids (e.g., ergovaline) and indole-diterpenes (e.g., lolitrem B) are active against both vertebrate and invertebrate herbivores, whereas peramine and loline alkaloids specifically affect invertebrate herbivores (Schardl et al. 2013).

Tall fescue [*Schedonorus phoenix* (Scop.) Holub. ex. *Lolium arundinaceum* (Schreb.) S. J. Darbyshire, syn. *Festuca arundinacea* Schreb.] is an important agronomic cool-season grass with a world-wide distribution. This grass often hosts the systemic endophytic fungus, *Epichloë coenophiala* (Morgan-Jones & W. Gams) C.W. Bacon & Schardl [formerly *Neotyphodium coenophialum* (Morgan-Jones & W. Gams) Glenn, C.W. Bacon & Hanlin]. Tall fescue originated in Eurasia, where it can be found growing in low competition environments, such as roadsides and seashores. The species was introduced to US in the late 1800’s, and today is a widely distributed and important forage and turf grass in the eastern and middle United States and is less widely distributed in South America, New Zealand, and Australia (Hoveland 1993; Young et al. 2014).

‘Kentucky-31’ (KY31) was the first widely used tall fescue cultivar in the US (Young et al. 2014). Empirical evidence has demonstrated that the systemic and asymptomatic endophytic fungus made the cultivar particularly vigorous, highly resistant to herbivores, and tolerant to a wide range of environmental conditions (Clay and Holah 1999; Saikkonen 2000). The endophyte thus has promoted competitive dominance of infected grasses especially in stressful environments (Malinowski and Belesky 2000; Saikkonen et al. 2004, 2006).

It also is well documented that the *Epichloë* endophyte-infected KY31 tall fescue can cause health problems for grazing vertebrates, especially during long and hot summers (Bacon 1995; Young et al. 2013). Consumption of endophyte-produced alkaloids has been implicated directly in the onset of animal stress, specifically in the case of the ergot alkaloids (Foote et al. 2013; Young et al. 2013). Significant scientific effort has been spent trying to unravel the mechanisms associated with this animal response. Surprisingly, less attention has been paid to the variation in alkaloid production of the grass-endophyte symbiota caused by variable environmental conditions and to how alkaloid-environment relationships may be influenced by the genetic background of the host grass.

We examined how alkaloid production in wild tall fescues originating from Northern Europe, which are highly infected with *Epichloë* endophytes (ca. 90 %, Saari et al. 2010), and the widely used US cultivar KY31 depends on variable environmental conditions. Endophyte-infected wild grasses from three different origins around the Baltic Sea and the cultivar KY31 were planted in identical common garden experiments in two environments, Northern Europe and Kentucky, where soil nutrients and water were manipulated. We expected the cultivar KY31 to produce high levels of alkaloids, especially in high nutrient environments (Arechavalenta et al. 1992) that mimic the agronomic-type environment. Nutrient application should increase the production of fungal alkaloids because alkaloids are nitrogen-rich compounds and nitrogen is often limiting in the environment. We also predicted that overall alkaloid production would be lower in Northern Europe than in Kentucky since in the latter environment the grasses would be exposed to higher temperatures and drought during the summers (Belesky et al. 1989; McCulley et al. 2014).

## Methods and Materials

### Plant Material

Seeds from naturally occurring, wild tall fescue populations from three areas that were separated approx. 500 km from each other by the Baltic Sea were collected in August 2003. We collected seeds from 8, 9, and 6 populations, respectively, from the island of Åland (A), the island of Gotland (G), and the West Coast of Sweden (S), 10–50 individual plants from each population. Three seeds from each individual plant were stained (Saha et al. 1988), and endophyte infections were determined by microscopic examination. All the tall fescue populations in the study had seed borne *Epichloë* sp., and infection frequencies varied between 85 and 100 %. We combined all E- (uninfected) seeds from populations within each area (A, G, and S) and all E+ (infected) seeds from populations within each area. In addition to plants from these natural tall fescue populations, both infected and uninfected ‘Kentuky-31’ (KY31) cultivar seeds were obtained from the University of Kentucky. Half of all the seeds were shipped from Finland to Kentucky or vice versa, so that both study sites had identical sets of seeds. At both locations, tall fescue seeds were germinated on moist tissue paper in Petri-dishes and planted in individual pots in a greenhouse. When plants had 2–3 tillers, they were transplanted to field plots.

### Common Garden Experiments

Parallel field experiments were carried out in the fields of Turku Botanical Garden, University of Turku (60°26′0″N, 22°10′19″E), Finland and the University of Kentucky‘s experimental Eden Shale Farm (38°32′22″N, 84°44′24″W), Kentucky, USA. The field site in Finland is at the edge of the northern distribution range of natural tall fescue populations, while the Kentucky field site is in the zone of intensive tall fescue cultivation in the USA, also known as the ‘fescue belt’. Both experimental field sites had been in cultivation in the past and were tilled without nutrient application in the summer 2004, immediately preceding experimental establishment. The experimental areas were fenced to prevent large vertebrates (e.g., rabbits, deer) access; however, smaller vertebrates (e.g., voles) and invertebrates were able to move freely into the experimental area and among plants.

In the control plots, pH of the experimental soils were 6.7 and 5.9, total nitrogen 0.15 % and 0.14 %, phosphorus 7 and 14 mg/kg, potassium 132 and 81 mg, calcium 1800 and 2080 mg/kg, and magnesium 208 and 120 mg/kg for Finland and Kentucky sites, respectively.

Both experimental set-ups were randomized block designs consisting of 10 blocks, each divided to 4 plots. In August 2004, E+ plants from four origins (A, G, S, KY31) were randomly planted in each experimental plot about 0.5 m apart and 0.5 m from the edge of the plot. The space between the experimental plants was either mowed, hand weeded, or sprayed with herbicide two times during the growing season to prevent interspecific competition. E- plants of the same origin also were planted in the plots, but were not included in this study, because uninfected plants are not capable of producing alkaloids.

The four plots in each block were designated randomly to one of the four treatments: Control (C) - receiving only ambient rainfall and nutrients, Water treatment (W) - with 3 L of water applied to each plant separately three times a week from June to August in Finland, and in Kentucky twice per week at 3.8 L per plant at each watering from April to October using trickle emitters and an irrigation timer. In Finland, nutrient treatment (N) was 1 dl of N-P-K-fertilizer (Nurmen Y2, Kemira KnowHow) (N-P-K/20–6-6) applied two times during the growing season for each plant. In Kentucky fertilization consisted of 50 kg/ha of N/per application in form of urea. Water and nutrient treatments (WN) combined both water and nutrient applications. These experiments allowed us to compare the effects of water and nutrient treatments within the experiments in Finland and Kentucky. However, comparisons between the two experiments should be done with caution because the sites inevitably differ in terms of numerous abiotic and biotic factors.

In 2005, all the experimental plants were re-checked to verify their endophyte status. In July, we sampled a pseudostem from each plant for immunoblot assay detection using monoclonal antibodies specific to *Epichloë* (Phytoscreen Immunoplot Kit #ENDO7973, Agrinostics, Watkinsville, GA, USA). In addition, at the end of the summer, three seeds from each plant were stained (Saha et al. 1988), and endophytic infections were checked by microscopic examination. All plants included in this analysis were infected with *Epichloë* (*N* = 40, 38, 40, 34 and *N* = 35, 38, 40, 38 for A, G, S, and KY31 origins in Finland and Kentucky experiments, respectively).

The phenological timing of the two experiments is difficult to compare, and therefore we decided to sample regrowth leaf biomass of the experimental plants. The samples were collected from spring regrowth and fall regrowth in Finland and Kentucky, respectively. We obtained samples from all the 152 E+ plants in the Finnish experiment in mid-June 2006. A handful of green growth was cut at the height of 5 cm from the ground, placed into a plastic bag, and transported immediately to a deep-freezer (−80 C°). The samples were ground, and the ground plant material from Finland was sent to Kentucky for alkaloid analyses. In Kentucky, 151 E+ plants were sampled in early November 2006. The samples from both experiments were analyzed using the same protocol.

### Alkaloid Analyses

Pyrrolizidine alkaloids [*N*-acetylloline (NAL), *N*-formylloline (NFL), and *N*-acetylnorloline (NANL)] were extracted from powdered plant material with ethanol:methylene chloride (4:1, *v*/v) containing the internal standard quinoline and sodium bicarbonate following protocol of Blankenship et al. (2001). Individual alkaloids were resolved and quantified by gas chromatography equipped with FID detector. Chromatographic conditions were 15 m × 0.53 mm DB5 column with initial oven temperature of 70 °C increased to 160 °C at 45 min^-1^, held for 5 min, and increased to 290 °C at 45 min^-1^ and held for 7 min.

Ergovaline, ergovalinine, and lysergic acid were measured by a modification of the procedure of Yates and Powell (1988). The HPLC was equipped with a fluorescence detector with excitation at 310 nm and measurement at 420 nm. Separation was on an Alltech Alltima C18 150 × 4.6 mm column with 3 μ particle size. Elution solutions were (A) 75 mM ammonium acetate in water:acetonitrile (3:1, *v*/v) and (B) acetonitrile. Elution gradient was 95:5 (A:B) for 1 min; linear change to 60:40 (A:B) during the next 15 min and maintained for 5 min; changed to 0:100 (A:B) during the next 1.5 min and maintained for 5 min; changed to 100:0 (A:B) during the next 1 min and maintained for 6 min before returning to 95:5.

### Statistical Analyses

Statistical analyses were performed using R 2.15.2 statistical software (R Core Team 2012) by applying linear mixed models (Pinheiro et al. 2013). The experiments conducted in Finland and Kentucky were analyzed separately because the sites inevitably differ in terms of numerous abiotic and biotic factors.

Total ergot alkaloids and lysergic acids were used as the final response variables, as results based on individual compounds did not differ from models based on their sums. Lolines were analyzed as sums of *N*-acetylloline (NAL), *N*-formylloline (NFL), and *N*-acetylnorloline (NANL). Normality of the residuals was gained after transformation as suggested by the Box-Cox analysis. All ergot alkaloids were log transformed (λ = 0), and to take into account zero values, one was first added to each compound before transformation. Lysergic acids were square root transformed (λ = 0.5). There was no need for transformation in lolines.

The statistical model for alkaloids included the fixed factors fertilization (N) and water treatment (W) treatments (included in models as, “N”, nitrogen and nitrogen + water combined, “W”, control and water treatment combined, and interaction, “N*W” between N and W), plant origin (KY31, A, G, S) and interactions between treatments and origin. Block was used as a random factor in all models. Association between different compounds within treatments (N and W) and origins were analyzed in separate linear mixed models (i.e., comparison of ergot totals vs. loline totals in nitrogen and control treatments for each origin).

The full model with treatments, origins, and their interactions was fitted, and the non-significant factors were removed. Out of these models, we constructed the final model by including all factors that were significant at least in one of the experiments. This model is shown for experiments conducted in each site. Interaction plots were used in illustrating the significant interactions between the factors left in the models.

## Results

All the 303 examined endophyte-infected tall fescue plants (152 from Finland and 151 from Kentucky) contained detectable amounts of ergot alkaloids and lolines. However, two S origin plants grown in Kentucky and one S and one KY31 origin plant grown in Finland did not produce lysergic acid.

### Ergot Alkaloids

**Fig. 1:**
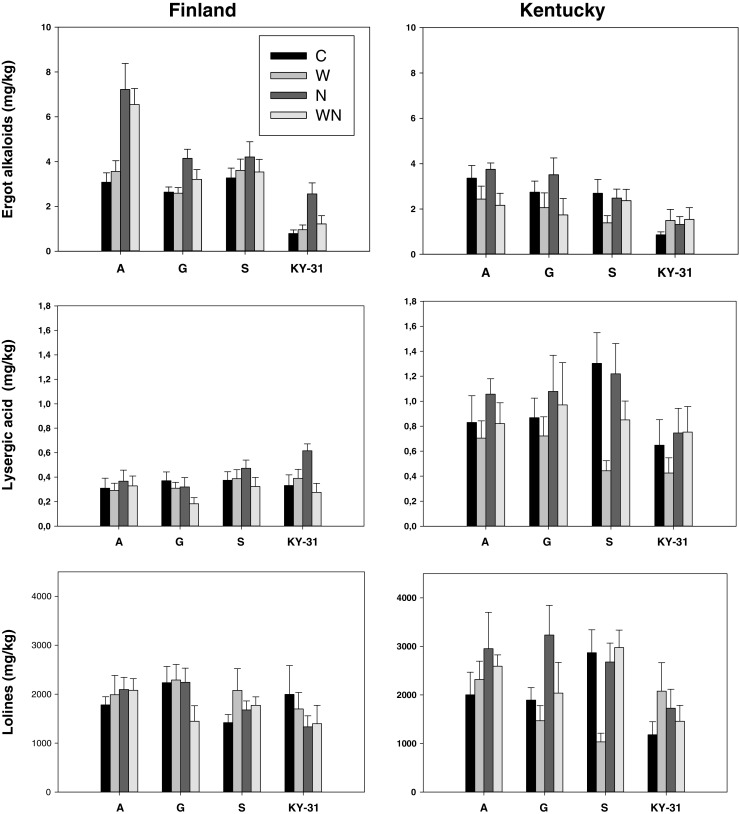
Total ergot alkaloid, lysergic acid and loline contents of endophyte-infected tall fescue plants from three wild populations and one cultivar (*A* Åland island, *G* Gotland island, *S* mainland Sweden, *KY-31* cultivar ‘Kentucky-31’) grown in common garden in Finland and Kentucky. The plants received either water (*W*), nutrient (*N*) or combined water and nutrient treatments (*WN*) while control plants (*C*) were left without extra water and nutrients (means ± SE)

Endophyte-infected tall fescue plants produced ca. 50 % more total ergot alkaloids in Finland compared to the plants with same origin and treatments that were grown in Kentucky (Fig. 1). Total ergot alkaloid concentrations were similar compared to ergovaline and ergovalinine concentrations in the two experiments, all origins and treatments.

KY31 plants produced equivalent amounts of total ergot alkaloids in common garden experiments at both locations at the time of the sampling (*t* = 0.25, *df* = 69, *P* = 0.80), while the wild origin plants produced more ergot alkaloids in Finland (*t* = 6.0, *df* = 211, *P* < 0.001). In Finland, the ergot alkaloid production by A origin plants was four-fold greater, and by S or G origin plants was three-fold greater, than production by KY31 plants (Fig. 1). In the Kentucky experiment, the wild origin plants (S, G, A) produced twice as much ergot alkaloids as the KY31 plants (Fig. 1).

**Fig. 2:**
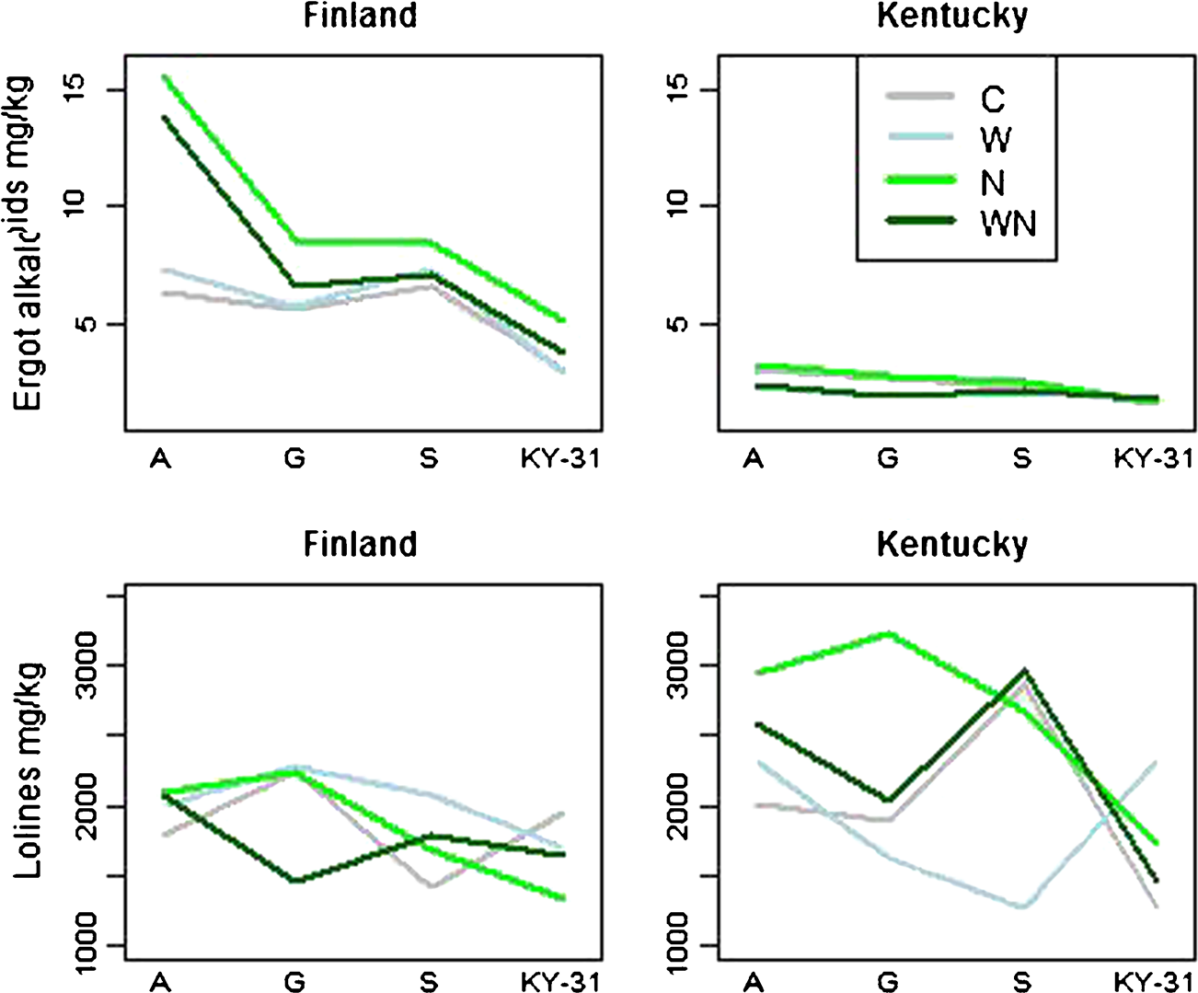
Model-based (Table 1) estimated means of treatments (*C* control, *W* water treatment, *N* nutrient treatment, *WN* water and nutrient treatment) for the endophyte-infected tall fescue plants from three wild populations and one cultivar (*A* Åland island, *G* Gotland island, *S* mainland Sweden, *KY-31* cultivar ‘Kentucky-31’) for total ergot alkaloids and lolines in plants grown in Finland or Kentucky. Note that separate models were used for Finland and Kentucky

Nutrition treatment tended to increase the amount of ergot alkaloids in Finland, but not in Kentucky (Table 1). Addition of nutrients was especially effective on A origin plants, which produced more than twice as much ergot alkaloids in nutrient (N) and combined nutrient and water (WN) treatments compared to control (C) and water (W) treatments in Finland (Fig. 2). Overall ergot alkaloid levels were lowest in the KY31 origin plants both in Finland and in Kentucky. Thus, KY31 expressed the lowest plasticity in response to nutrient and water treatments. Of the wild origin plants, A plants showed the highest plasticity followed by G and S origin plants (Fig 2., Table 1).

**Table 1:** Linear mixed models for total ergot alkaloids in tall fescue plants. The grasses from the wild origins (Orig) Gotland (G) and Sweden (S) and cultivar ‘Kentucky-31’ (KY31) were compared with wild origin Åland (*A*) in water (*W*) and nutrition (*N*) treatment (*Treat*) plots. Results for the same model are shown for plants grown in Finland or Kentucky with all main factors and those interactions that were statistically significant in either of the experiments. Model estimates are standard errors (*S.E.*), degrees of freedom (*df*), and *t*-tests (*t*) with *P*-values (*P*) *** < 0.001,** < 0.01,* < 0.05, o < 0.1

	FINLAND						KENTUCKY					
	Value	S.E.	*df*	*t*	*P*		Value	S.E.	*df*	*t*	*P*	
Intercept	1.980	0.104	103	19.04	0.001	***	2.065	0.130	100	15.85	0.001	***
Treat N	0.839	0.130	36	6.45	0.001	***	0.095	0.172	36	0.55	0.584	
Treat W	0.144	0.130	36	1.11	0.276		-0.286	0.165	36	-1.74	0.091	o
W*N	-0.262	0.123	36	-2.13	0.040	*	-0.064	0.165	36	-0.39	0.701	
Orig G	-0.092	0.139	103	-0.66	0.509		-0.122	0.163	100	-0.75	0.454	
Orig S	0.046	0.139	103	0.33	0.741		-0.278	0.159	100	-1.74	0.084	o
Orig KY31	-0.606	0.144	103	-4.20	0.001		-0.694	0.160	100	-4.34	0.001	***
N*G	-0.461	0.163	103	-2.84	0.006	**	-0.036	0.189	100	-0.19	0.849	
N*S	-0.619	0.160	103	-3.86	0.001	***	0.027	0.188	100	0.15	0.884	
N*KY31	-0.423	0.169	103	-2.50	0.014	*	0.011	0.189	100	0.06	0.953	
W*G	-0.133	0.163	103	-0.82	0.416		-0.035	0.189	100	-0.18	0.855	
W*S	-0.047	0.160	103	-0.29	0.772		0.151	0.188	100	0.8	0.423	
W*KY31	-0.176	0.170	103	-1.03	0.304		0.429	0.189	100	2.26	0.026	*
AIC	177.01						214.71					
loglikelihood	-73.50						-92.35

### Lysergic Acid

The total amount of lysergic acid, a precursor for a wide range of ergot alkaloids, was 60 % higher in tall fescue plants grown in Kentucky compared to those grown in Finland.

In Finland, treatments and plant origin affected lysergic acid concentrations, but the fit of the model is poor (log likelihood =33.4). In the Kentucky experiment, lysergic acid concentrations were significantly lower in KY31 plants compared to wild origin (A, G, S) plants (Table 2, model log likelihood = −43.1). Lysergic acid concentrations tended to be lower for water-treated plants.

**Table 2:** Linear mixed models for total lysergic acids and lolines in tall fescue plants grown in Kentucky. The grasses from the wild origins (*Orig*) Gotland (*G*) and Sweden (*S*) and cultivar ‘Kentucky-31’ (*KY31*) were compared with wild origin Åland (*A*) in water (*W*) and nutrition (*N*) treatment (*Treat*) plots. Results are shown for with all main factors and those interactions that were statistically significant. Model estimates are standard errors (*S.E.*), degrees of freedom (*df*), and *t*-tests (*t*) with *P*-values (*P*) *** < 0.001,** < 0.01,* < 0.05, o < 0.1

	LYSERGIC ACID					
	Value	S.E.	*df*	*t*	*P*	
Intercept	0.960	0.067	106	14.355	0.001	***
Treat W	-0.117	0.059	37	-1.967	0.057	o
Treat N	0.084	0.059	37	1.427	0.162	
Orig G	0.005	0.067	106	0.073	0.941	
Orig S	0.051	0.067	106	0.762	0.448	
Orig KY31	-0.142	0.067	106	-2.106	0.038	*
AIC	102.17					
loglikelihood	-43.09					
	LOLINES					
	Value	S.E.	*df*	*t*	*P*	
Intercept	42.354	4.426	94	9.569	0.001	***
Treat W	11.330	6.963	36	1.627	0.112	
Treat N	4.341	6.109	36	0.711	0.482	
W*N	-7.556	9.161	36	-0.825	0.415	
Orig G	0.114	5.808	94	0.019	0.984	
Orig S	9.512	5.641	94	1.686	0.095	o
Orig KY31	-8.499	5.807	94	-1.463	0.147	
N*G	-0.066	8.652	94	-0.008	0.994	
N*S	-13.004	8.541	94	-1.523	0.131	
N*KY31	-4.916	8.652	94	-0.568	0.571	
W*G	-7.875	8.098	94	-0.972	0.333	
W*S	-20.203	8.125	94	-2.486	0.015	*
W*KY31	8.132	8.420	94	0.966	0.337	
N*W*G	-1.509	11.770	94	-0.128	0.898	
N*W*S	26.624	11.709	94	2.273	0.025	*
N*W*KY31	-8.947	11.916	94	-0.751	0.455	
AIC	1109.56					
loglikelihood	-536.78					

### Loline Alkaloids

The amount of total lolines was 17 % lower in plants grown in Finland compared to plants grown in Kentucky. In Finland, neither nutrients nor water application affected loline alkaloid concentrations (Fig. 2). In the Kentucky experiment, overall total loline concentrations (*N*-acetylnorloline, *N*-formyloline, *N*-acetyloline) of different plant origins were affected in different ways by different treatments causing high level interaction between the predictors (Fig. 1, Table 2).

### Correlations Between Alkaloids

In both the Finnish and the Kentucky experiments, different components within the three chemical groups, ergot alkaloids, lysergic acids, and lolines, showed high positive correlations (the lowest correlation in Finland was *r* = 0.67 and in Kentucky *r* = 0.87) when all the treatments were included in the analysis. This indicates that e.g., a plant that produces high concentrations of ergot alkaloids also produces high concentrations of lysergic acid and lolines. Correlations between means of the three groups were higher in plants grown in Kentucky (correlations between 0.53 and 0.65) compared to those grown in Finland (correlations between 0.23 and 0.31). All these correlations were statistically significant. Correlations among individual chemicals were also higher in Kentucky plants (range *r* = 0.45 to 0.67) compared to Finland plants (range *r* = 0.08 to 0.45).

## Discussion

In contrast to our prediction that KY31 should produce more ergot alkaloids than its wild population counterparts, KY31 endophyte-infected plants produced 2–4 times lower ergot alkaloid concentrations compared to plants from wild European populations when the plants were grown in the same environmental conditions. The lower ergot alkaloid production by KY31 was evident regardless of the experimental site (Finland or Kentucky) and the various water and nutrient treatments. We can conclude thus that the most widely grown tall fescue cultivar in US has genetically relatively low ability to produce ergot alkaloids compared to wild tall fescue plants from Northern Europe. This implies that the success of endophyte-infected tall fescue KY31 in North America and other areas has not been due to unusually high ergot alkaloid production under high herbivore pressure. Rather, success of KY31 tall fescue must be based upon some other traits associated with the symbiotum. The original selection criteria of the tall fescue cultivar KY31 were most likely its excellent agronomic attributes under drought, poor soils, and intense grazing (Saikkonen et al. 1998, 2004; Young et al. 2014).

Comparing results from intercontinental reciprocal transplantation experiment is challenging because uncontrolled abiotic and biotic factors inevitably differ between continents. For example, differences in soil types, timing and length of growing season, seasonal changes in weather conditions and day length are likely to affect alkaloid concentrations in the endophyte-grass symbiotum. However, tall fescue and particularly its cultivar KY31 is a unique baseline model for our study. Chemotypic diversity of tall fescue is well documented in the US (Siegel and Bush 1996), including ample evidence of seasonal changes in alkaloid production across years (McCulley et al. 2014; Repussard et al. 2014). Thus, deviations from norms can be distinguished and linked to the explanatory or experimental factors.

For example, our results demonstrate clearly that wild grasses produced high amounts of ergot alkaloids in both study sites, whereas ergot alkaloid production of KY31 remained relatively low. In their five year field study, McCulley et al. (2014) observed total ergot alkaloid concentrations range between 0.4–1.9 mg/kg in KY31 tall fescue cultivar. This is in accordance with ergot alkaloid production by KY31 when grown in Finland or Kentucky (0.9–1.1 mg/kg) in the present study. However, the wild origin plants produced 3–5 and 2–3 times higher amounts of ergot alkaloids in Finland and in Kentucky, respectively. In addition, all ergovaline concentrations in our samples substantially exceeded the toxicity threshold value (0.4 mg/kg) known for cattle (Repussard et al. 2014). Thus, our results provide solid evidence for the potential of infected wild tall fescue genotypes to produce high levels of alkaloids with possible injurious effects on animal husbandry compared to KY31.

In contrast to recent experimental evidence by McCulley et al. (2014) that warming markedly increases concentrations of ergot alkaloids in tall fescue, our study found that levels of ergot alkaloids were higher in Finland than in the US where growing seasons are slightly warmer. This suggests that temperature alone fails to explain the alkaloid concentrations in endophyte-infected fescue. Higher concentrations of lysergic acid in plants growing in Kentucky suggest that the plants had high potential for ergot alkaloid production because lysergic acid is a precursor of ergot alkaloids (Schardl et al. 2013). Interestingly, in Finland the lysergic acid concentrations in plants with different origins were the same, while ergot alkaloid concentrations were higher in wild origin plants compared to KY31. This indicates that plant genotype x environment-interaction may play a significant role in the ergot alkaloid pathway.

McCulley et al. (2014) suggested that alkaloid production of lolines is boosted by combined effects of higher temperature and precipitation. Our results support this idea - concentrations of lolines were higher in plants grown in Kentucky. However, S origin plants produced low concentrations of lolines when they were treated with water, but high concentrations of lolines with the other treatments, consistent with the possible genotype x environment interaction in loline production in tall fescue plants.

As a testable hypothesis for future studies, we suggest that alkaloid production in certain grass-fungal genotype combinations is affected by combined effects of nutrient availability in soils, temperature, precipitation, and day length, which determine the energy available for photosynthesis. For example, shorter growing season but longer day length during the growing season in Finland, due to higher latitudes (Saikkonen et al. 2012), may promote photosynthesis and, accordingly, carbon resources in a plant. Alkaloids are nitrogen-based compounds and it has been suggested that harboring the systemic endophyte is costly to the plant when nutrients are the limiting factor in the soil (Ahlholm et al. 2002; Lehtonen et al. 2005; Saikkonen et al. 2006). Highly competitive fungal genotypes may reap benefits of photosynthate surplus in plants growing in long day conditions, but alkaloid production of fungus can be limited by nitrogen availability in soils. Thus, certain fungal genotypes may accumulate high amount of mycelia in the host plant but produce higher amount of alkaloids only when fertilized. Well replicated experimental studies with endophyte-plant genotype-genotype combinations coupled with quantifying mycelial biomass and alkaloid production in plants growing in differently fertilized soils are necessary to test this hypothesis. These results could be applied into grass breeding programs aiming to develop cultivars for different environments and purposes (Gundel et al. 2013; Saikkonen et al. 2015).
